# Dry needling, lidocaine, and mepivacaine for myofascial pain and associated temporomandibular disorder and depressive symptoms: a randomized clinical trial

**DOI:** 10.1186/s12903-026-08986-0

**Published:** 2026-06-22

**Authors:** Sümer Münevveroğlu, Duygu Başeğmez, Hasan Burak Karaca

**Affiliations:** 1 Department of Oral and Maxillofacial Surgery, Faculty of Dentistry, Istanbul Medipol University, Istanbul, Turkey; 2 Graduate School of Health Sciences, Department of Oral and Maxillofacial Surgery, Istanbul Medipol University, Istanbul, Turkey

**Keywords:** Myofascial pain, trigger point injection, dry needling, lidocaine, mepivacaine, temporomandibular disorder

## Abstract

**Background:**

Myofascial pain of the masticatory muscles significantly contributes to temporomandibular disorders (TMD) and is often associated with impaired function and depressive symptoms. While trigger point injections like dry needling and local anesthetics are common, direct comparisons in the orofacial region are limited. This randomized trial compared the effectiveness of dry needling, lidocaine, and mepivacaine regarding myofascial pain, self-reported TMD symptoms, and depressive symptoms.

**Methods:**

In this double-blind trial, 75 patients with bilateral active masseter and temporalis trigger points were randomized to dry needling, lidocaine (20 mg/mL), or mepivacaine (30 mg/mL) (*n* = 25 each). Interventions were administered weekly for three sessions. Pain intensity (Visual Analog Scale), depressive symptoms (Beck Depression Inventory), and TMD symptoms (Fonseca Anamnestic Index) were assessed at baseline and at a short-term follow-up (one week post-treatment). Two-way repeated measures ANOVA with Bonferroni post-hoc tests evaluated interactions between time and treatment.

**Results:**

All interventions significantly reduced pain, depressive, and TMD scores (*p* < 0.001). A significant time x group interaction was observed for pain intensity (*p* < 0.001). Mepivacaine achieved the greatest mean pain reduction (5.36), which was significantly superior to dry needling (*p* = 0.001), whereas no significant difference was found between lidocaine and dry needling (*p* = 0.226). No significant between-group differences were observed for changes in depressive or TMD symptom scores (*p* > 0.05).

**Conclusions:**

All three modalities effectively reduced myofascial pain and improved psychological outcomes within the short-term observation period of one week. Mepivacaine demonstrated superior short-term pain reduction compared to dry needling. Both local anesthetics represent viable options for short-term myofascial pain management, though studies with longer follow-up periods are required to evaluate sustained effects and comparative advantages.

**Trial registration:**

ClinicalTrials.gov, NCT07079449. Registered on July 14, 2025.

## Background

Myofascial pain syndrome is a common musculoskeletal disorder characterized by the presence of hyperirritable trigger points within taut bands of skeletal muscle fibers [1]. This condition is frequently identified in the orofacial region, particularly within the masseter and temporalis muscles, and is documented as the most prevalent subtype of temporomandibular disorders (TMD) [2]. Patients with myofascial pain syndrome typically present with localized pain, restricted mandibular movement, and referred pain patterns. These symptoms can significantly impair quality of life and are often associated with psychosocial comorbidities such as depressive symptoms [3, 4].

Trigger point injections are widely employed in the management of myofascial pain syndrome [5, 6]. Dry needling exerts therapeutic effect through mechanical stimulation, which may induce a local inflammatory response and enhance regional microcirculation [7]. In contrast, local anesthetics such as lidocaine and mepivacaine, when administered without vasoconstrictors, produce vasodilation and improve perfusion through pharmacologic mechanisms [8]. Despite their differing modes of action, all three modalities aim to deactivate trigger points and support tissue recovery.

Although these approaches demonstrate clinical efficacy, only a few studies have directly compared their effectiveness in the masticatory muscles [9, 10]. Moreover, while the primary goal of trigger point injections is pain reduction, their impact on broader clinical outcomes such as self-reported TMD symptoms and depressive symptoms remains underexplored. Assessing these domains may offer a more comprehensive view of each intervention’s therapeutic potential.

Therefore, the aim of this randomized clinical trial was to compare the effects of dry needling, lidocaine, and mepivacaine trigger point injections on myofascial pain in the masseter and temporalis muscles, and to evaluate their impact on self-reported TMD symptoms and depressive symptoms. Direct comparative evidence in the masticatory muscles remains limited, leaving clinicians without clear guidance on selecting the most effective injection modality. Although lidocaine is widely used, its pronounced vasodilatory effect can lead to rapid systemic absorption. In contrast, mepivacaine induces significantly less vasodilation, allowing for prolonged local retention, and possesses a pKa closer to physiological pH, which better maintains its lipid-soluble form in acidic inflamed tissues [11, 12]. Given these pharmacological advantages, we hypothesized that while all three interventions would improve symptoms, mepivacaine would offer the greatest analgesic benefit. In clinical practice, a reduction of approximately 1.5 to 2.0 cm on a 10-cm visual analog scale is generally considered a meaningful magnitude of clinical benefit for patients suffering from temporomandibular and orofacial pain [13]. By systematically evaluating pain, function, and psychological outcomes, this trial was designed to provide clinically relevant data to inform the patient-centered management of myofascial pain.

## Methods

### Study design and participants

This double-blind, randomized clinical trial was conducted at the Faculty of Dentistry, Istanbul Medipol University, in accordance with the Declaration of Helsinki. Ethical approval was obtained from the university’s ethics committee (Approval No: E-10840098-202.3.02-2739), and the study was registered at ClinicalTrials.gov (ID: NCT07079449) on July 14, 2025.

A total of 138 patients presenting with orofacial pain were screened for eligibility between May and August 2025. Of these, 63 patients were excluded: 54 did not meet the specific diagnostic criteria for myogenous pain (e.g., presenting with unilateral pain, an insufficient number of active trigger points, or primarily arthrogenous TMD) and 9 declined to participate, resulting in a final enrollment of 75 participants. The study cohort consisted of 60 females (80%) and 15 males (20%). Inclusion criteria were as follows: adults aged 18 to 64 years; the presence of bilateral active myofascial trigger points in both the masseter and temporalis muscles; a minimum of three active trigger points in the masseter and at least one in the temporalis muscle; and willingness to provide written informed consent. Active trigger points were identified based on international consensus criteria [14], defined by the presence of a hypersensitive spot within a taut muscle band, coupled with the reproduction of the patient’s familiar pain upon sustained digital pressure of approximately 1 kg/cm² [15]. To ensure consistency, the examiner was calibrated to apply approximately 1 kg of pressure using a digital scale prior to patient evaluations. Exclusion criteria included: a history of prior or ongoing treatment for TMD; systemic neuromuscular diseases; pregnancy or breastfeeding; use of analgesics, muscle relaxants, or antidepressants within the preceding two weeks; and the presence of active infection, trauma, or tumors in the orofacial region.

### Interventions and blinding

Participants were randomly allocated to one of three treatment groups (dry needling, lidocaine injection, or mepivacaine injection) using a computer-generated randomization sequence created by an independent researcher not involved in clinical assessments or treatments. Allocation concealment was ensured using sequentially numbered, opaque, sealed envelopes.

Each participant received three treatment sessions at one-week intervals. All procedures were performed using 32-gauge, 13-mm needles to ensure standardization. The interventions followed the fanning technique described by Hong [16]. Upon needle insertion into the active trigger point, the elicitation of either a local twitch response or distinct localized hypersensitivity served as clinical confirmation of accurate needle placement. Immediately upon this confirmation, the intervention was applied directly into that specific locus. In the injection groups, a standard clinical volume of 0.2 mL of either plain lidocaine (20 mg/mL) or mepivacaine (30 mg/mL) without vasoconstrictors was administered per trigger point at each session. Injections were applied to a minimum of three and a maximum of five sites in the masseter muscle (up to 1.0 mL total per side), and one or two sites in the temporalis muscle (up to 0.4 mL total). In the dry needling group, the needle was manipulated using the identical fanning technique to ensure a comparable mechanical sensation, without any fluid injection.

A rigorous protocol was established to maximize blinding. The outcome assessor, who conducted all pre-treatment and post-treatment evaluations, was strictly blinded to group allocation. Participant blinding was inherently supported by the standard clinical protocol; the routine intramuscular administration of a minimal anesthetic volume (0.2 mL per site) naturally limits cutaneous paresthesia. Consequently, participants did not experience a distinct surface numbing sensation that would easily unblind them. Additionally, the mechanical intramuscular manipulation during dry needling closely mimicked the pressure sensation of the injection procedure. While it is a methodological limitation that the treating clinician could not be blinded to the distinction between dry needling and fluid injection, strict blinding was maintained regarding the type of anesthetic agent. For the injection groups, a clinical nurse prepared either lidocaine or mepivacaine in identical, unmarked syringes according to the concealed allocation sequence immediately prior to the procedure, ensuring the injecting clinician remained completely blinded to the specific pharmacological agent administered.

Regarding concomitant care, no additional analgesics, muscle relaxants, or adjunctive physical therapies were prescribed during the entire study period. Following the verification of the pre-operative exclusion criteria regarding prior analgesic use, patients were instructed to contact the clinic for an unscheduled control visit if they experienced an exacerbation of pain requiring rescue medication. Notably, no participants reported the use of supplemental painkillers throughout the trial, nor did any patient require an additional clinical visit for pain management.

### Outcome measures

The primary outcome measure was pain intensity, assessed with a 10-cm visual analog scale (VAS). To represent the overall clinical pain intensity and to ensure statistical independence, the VAS scores from the right and left sides were averaged to calculate a single mean VAS score for each participant at each time point. Secondary outcomes included depressive symptoms, evaluated by the Beck Depression Inventory, and self-reported temporomandibular disorder symptom scores, assessed using the Fonseca Anamnestic Index. The Fonseca Anamnestic Index was selected for this study due to its proven reliability and high correlation with objective clinical indices, such as the Helkimo Index and Diagnostic Criteria for Temporomandibular Disorders, making it an effective instrument for monitoring clinical progression and symptom burden in longitudinal studies [17, 18]. All assessments were conducted at baseline and one week after the third and final treatment session.

### Statistical analysis

The sample size was determined using a power analysis performed with G*Power version 3.1.9.7 (Heinrich-Heine-Universität Düsseldorf, Düsseldorf, Germany). Based on a predicted medium effect size (f = 0.25) for the primary outcome (mean VAS), with an alpha level of 0.05 and a power of 0.80 for a Two-way Repeated Measures ANOVA, a total sample size of at least 66 participants was required. To account for potential dropouts and ensure robust analysis, 75 participants (25 per group) were enrolled in the study.

Statistical analyses were performed using IBM SPSS Statistics version 27 (IBM Corporation, Armonk, NY, USA) in accordance with the intention-to-treat (ITT) principle. Since no participants dropped out during the study, the ITT population was identical to the per-protocol population (*n* = 75). The Shapiro–Wilk test was used to confirm the normality of the data distribution. Baseline demographic data, including age and sex, were compared between the three treatment groups using one-way analysis of variance and the Pearson chi-square test, respectively. For the primary outcome, which was the mean visual analog scale score, and the secondary outcomes, including the Beck Depression Inventory and the Fonseca Anamnestic Index scores, a Two-way Repeated Measures ANOVA was utilized. This model included “Treatment Group” (dry needling, lidocaine, and mepivacaine) as the between-subjects factor and “Time” (baseline and post-treatment) as the within-subjects factor. The interaction effect between treatment group and time was specifically analyzed to determine the differential efficacy of the interventions. Post hoc pairwise comparisons were performed using the Bonferroni correction to identify specific differences while controlling for Type I error. To provide a high level of analytical precision, effect sizes (partial eta squared, $\:{\eta\:}_{p}^{2}$) and 95% confidence intervals were calculated for all outcomes. A p-value of less than 0.05 was considered statistically significant.

## Results

**Fig. 1:**
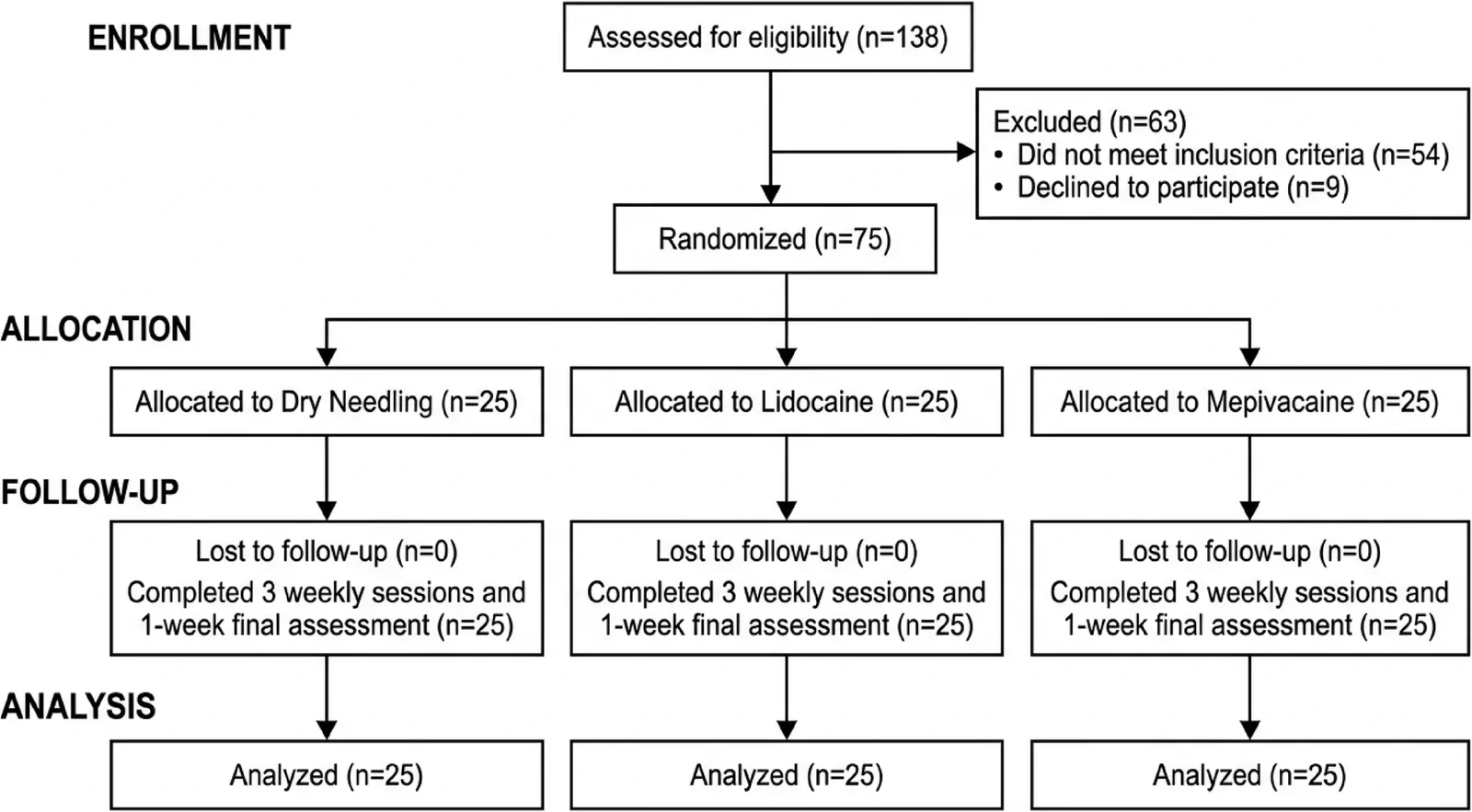
CONSORT flow diagram of the study participants, detailing the enrollment, allocation, follow-up, and analysis phases

Seventy-five patients were enrolled and randomly allocated to three equal treatment groups (*n* = 25 each): dry needling, lidocaine injection, and mepivacaine injection. Participant flow through the study is detailed in the CONSORT flow diagram (Fig. 1). No participants were lost to follow-up, and all completed the study protocol without adverse events or missing data.

The overall mean age of participants was 26.2 ± 5.9 years. Similarly, sex distribution was balanced across groups, with 60 females (80%) and 15 males (20%) overall. There were no statistically significant differences between groups regarding age or sex distribution (*p* > 0.05 for both), confirming the successful randomization and baseline homogeneity of the study population (Table 1).

**Table 1:** Demographic characteristics of the study groups

Group	Mean Age (years) ± SD	*n*	Female (%)	Male (%)	*p*-value
Dry Needling	26.4 ± 6.2	25	21 (84%)	4 (16%)	
Lidocaine	25.9 ± 5.8	25	19 (76%)	6 (24%)	
Mepivacaine	26.3 ± 5.7	25	20 (80%)	5 (20%)	
Total	26.2 ± 5.9	75	60 (80%)	15 (20%)	0.963^a^ / 0.779^b^

^a^One-way ANOVA for age; ^b^ Pearson chi-square test for sex distribution

### Pain intensity

All three interventions resulted in statistically significant reductions in facial pain intensity, as measured by the mean VAS scores. Two-way repeated measures ANOVA revealed a significant main effect of time (*p* < 0.001, $\:{\eta\:}_{p}^{2}$ = 0.795), indicating that pain intensity decreased significantly in all participants regardless of the treatment group. Furthermore, a significant time x group interaction was observed (*F*(2, 72) = 18.184, *p* < 0.001, $\:{\eta\:}_{p}^{2}$ = 0.336), demonstrating that the efficacy of the interventions differed significantly across the three groups over the follow-up period. Post hoc pairwise comparisons with Bonferroni correction revealed that the mepivacaine group achieved the greatest mean reduction in pain intensity (5.36), followed by the lidocaine (3.62) and dry needling groups (2.10). Notably, the mepivacaine group was the only intervention to demonstrate statistical superiority over the dry needling group at the post-treatment follow-up (*p* = 0.001), whereas the difference between the lidocaine and dry needling groups did not reach statistical significance (*p* = 0.226). Although the mepivacaine group showed a higher mean reduction compared to the lidocaine group, this specific difference was not statistically significant (*p* = 0.125), yet it represented a favorable clinical trend toward mepivacaine. Detailed mean VAS scores and 95% confidence intervals are presented in Table 2.

**Table 2:** Longitudinal analysis of mean VAS scores and treatment efficacy

Group (*n* = 25 each)	Baseline (Mean ± SD) [Min-Max]	Post-treatment (Mean ± SD) [Min–Max]	Mean Change (95% CI) [Min–Max]	Partial Eta Squared ($\:{\eta\:}_{p}^{2})$
Dry Needling	5.98 ± 0.90 [5.5–9.0]	3.88 ± 0.82 [2.5–5.5]	2.10 (1.75 − 2.45) [1.0–3.5]	0.21
Lidocaine	6.56 ± 0.88 [5.0–8.5]	2.94 ± 0.75 [1.5–4.5]	3.62 (3.22 − 4.02) [2.5–5.0]	0.34
Mepivacaine	7.22 ± 0.92 [5.5–9.0]	1.86 ± 0.68 [0.0–3.0]	5.36 (4.98 − 5.74) [4.0–7.0]	0.42
RM-ANOVA Panel	Source of Variation	*F*-statistic (*df* 2, 72)	*p*-value	$\:{\eta\:}_{p}^{2}$(Total)
	Interaction (Time × Group)	18.184	< 0.001	0.336

*SD* Standard Deviation, *CI* Confidence Interval, *VAS* Visual Analog Scale. All scores represent the mean VAS values, calculated by averaging measurements from the right and left sides. The RM-ANOVA Panel evaluates the interaction between Time and Treatment Group. Post-hoc pairwise comparisons with Bonferroni correction indicated that the mepivacaine group was the only intervention to demonstrate statistical superiority over the dry needling group at the post-treatment assessment (*p* = 0.001). No significant difference was found between the lidocaine and dry needling groups (*p* = 0.226) or between the mepivacaine and lidocaine groups (*p* = 0.125)

### Depressive symptoms

Regarding depressive symptoms, two-way repeated measures ANOVA revealed a significant main effect of time for Beck Depression Inventory scores (*p* < 0.001, $\:{\eta\:}_{p}^{2}$ = 0.512), indicating that all three interventions were effective in reducing depressive symptoms over the follow-up period. However, no significant time x group interaction was observed (*F*(2, 72) = 1.192, *p* = 0.309, $\:{\eta\:}_{p\:}^{2}$= 0.032). Although the mepivacaine group showed the greatest numerical reduction in mean scores (5.96), followed by the lidocaine (5.28) and dry needling groups (3.96), these differences between groups did not reach statistical significance.

### Self-reported temporomandibular disorder symptoms

All groups demonstrated significant improvement in self-reported TMD symptoms, as assessed by the Fonseca Anamnestic Index. Two-way repeated measures ANOVA showed a significant main effect of time (*p* < 0.001, $\:{\eta\:}_{p\:}^{2}$ = 0.584), but no significant time x group interaction was found (*F*(2, 72) = 1.303, *p* = 0.278, $\:{\eta\:}_{p\:}^{2}$ = 0.035). Descriptively, the mepivacaine group showed the greatest reduction in mean scores (15.60), followed by the lidocaine (13.08) and dry needling groups (12.72). Detailed results for these parameters, including mean scores and intervals, are presented in Table 3.

**Table 3:** Analysis of beck depression inventory and fonseca anamnestic index scores

Outcome Measure & Group (*n* = 25 each)	Baseline (Mean ± SD) [Min–Max]	Post-treatment (Mean ± SD) [Min–Max]	Mean Change (95% CI) [Min–Max]	Partial Eta Squared ($\:{\eta\:}_{p}^{2}$)
Beck Depression Inventory				
Dry Needling	14.20 ± 2.8 [10–19]	10.24 ± 2.5 [6–15]	3.96 (3.30–4.62) [2–6]	0.51
Lidocaine	14.85 ± 3.1 [10–21]	9.57 ± 2.4 [5–14]	5.28 (4.70–5.86) [3–8]	0.54
Mepivacaine	15.10 ± 2.9 [11–20]	9.14 ± 2.2 [5–13]	5.96 (5.42–6.50) [4–8]	0.58
RM-ANOVA Result (Interaction)	*F*(2, 72) = 1.192	*p* = 0.309	$\:{\eta\:}_{p}^{2}$ = 0.032	
Fonseca Anamnestic Index				
Dry Needling	58.40 ± 6.5 [45–70]	45.68 ± 5.8 [35–55]	12.72 (11.7–13.7) [8–18]	0.55
Lidocaine	60.15 ± 7.2 [45–75]	47.07 ± 6.4 [35–60]	13.08 (11.9–14.2) [9–20]	0.56
Mepivacaine	62.30 ± 6.8 [50–75]	46.70 ± 5.5 [35–55]	15.60 (14.4–16.8) [10–22]	0.62
RM-ANOVA Result (Interaction)	*F*(2, 72) = 1.303	*p* = 0.278	$\:{\eta\:}_{p}^{2}$ = 0.035	

*SD* Standard Deviation, *CI* Confidence Interval. All baseline and post-treatment comparisons for within-group changes were statistically significant (*p* < 0.001) for all interventions. The RM-ANOVA Result (Interaction) evaluates whether the treatment groups differed significantly in their rate of improvement over time. No significant interaction was found for either depressive symptoms (*p* = 0.309) or self-reported TMD symptoms (*p* = 0.278)

## Discussion

This randomized clinical trial compared the effects of dry needling, lidocaine injection, and mepivacaine injection on myofascial pain in the masseter and temporalis muscles, alongside their influence on self-reported TMD symptoms and depressive symptoms. All three interventions significantly reduced pain intensity, with the mepivacaine group showing the greatest improvement. Additionally, while improvements were observed in self-reported TMD symptom and depressive symptom scores across all groups, the differences between groups in these secondary outcomes were not statistically significant.

Mepivacaine demonstrated the greatest numerical reduction in pain intensity among the three interventions evaluated in this trial, although the difference compared to lidocaine was not statistically significant. This trend is consistent with its pharmacologic properties, which may hypothetically contribute to prolonged local action and enhanced analgesic effects. Albagieh et al. previously reported reduced post-injection tenderness with mepivacaine compared to lidocaine in patients with orofacial myofascial pain [19]. However, their study focused on immediate post-procedural sensitivity at the injection site, whereas our trial assessed sustained pain relief following multiple sessions. These methodological and outcome differences may limit direct comparability, yet both studies highlight the potential clinical utility of mepivacaine.

Although lidocaine is known to exert stronger vasodilatory effects in vivo, mepivacaine induces significantly less vasodilation—potentially resulting in slower systemic absorption and prolonged local retention at the injection site [11]. Moreover, in mildly acidic conditions such as inflamed tissue, mepivacaine retains effective analgesia due to its pKa proximity to physiological pH, which helps preserve its lipid-soluble form and tissue penetration [12]. While our study design precludes drawing direct mechanistic conclusions, these pharmacodynamic properties provide a plausible theoretical framework that aligns with the pronounced numerical reductions in pain intensity observed in the mepivacaine group. Importantly, the magnitude of pain reduction achieved across all intervention groups exceeded the established minimal clinically important difference (MCID) of 1.5 to 2.0 cm for orofacial pain [13]. This indicates that the statistically significant findings observed in our trial successfully translated into meaningful symptomatic relief for the patients.

In addition to significant pain reduction, all treatment modalities resulted in meaningful improvements in self-reported TMD symptom and depressive symptom scores. However, these secondary outcomes did not differ significantly among groups. The reduction in Fonseca Anamnestic Index scores observed in our study reflects a meaningful decrease in the overall symptom burden experienced by patients. While the Fonseca Anamnestic Index is not a substitute for the Diagnostic Criteria for Temporomandibular Disorders (DC/TMD) in establishing a primary diagnosis, its sensitivity to clinical changes makes it a valid parameter for evaluating treatment efficacy and functional improvement over time [20]. This pattern suggests that functional and psychological improvements likely reflect overall pain reduction rather than specific pharmacological effects. Previous studies confirm the strong association between chronic orofacial pain and psychological comorbidities, with prevalence rates of clinically significant anxiety and depression in TMD patients reported as high as 40–60% [21, 22]. Similarly, some researchers have linked pain severity directly to diminished emotional well-being and mandibular dysfunction [23, 24]. Although the parallel improvements in pain, mood, and function observed in our study are consistent with this concept, the current study design precludes establishing a direct causal relationship. Therefore, while it is plausible that decreased chronic nociceptive input contributes to improved emotional well-being, these secondary findings should be interpreted with caution.

The predominance of female participants in our study (80%) aligns with previous reports indicating a higher prevalence of myofascial pain and TMD among women. Several studies have demonstrated that women are more likely to develop myofascial pain, potentially due to hormonal, psychological, and neuromuscular differences [2, 25, 26]. This gender distribution should be considered when interpreting the generalizability of the results, as pain perception, treatment response, and associated psychological factors may differ by sex.

The lack of participant attrition—likely attributable to the short-term follow-up period and the institutional protocol of conducting telephone reminders prior to each appointment—along with the use of standardized, validated outcome measures, significantly enhances the internal validity of our findings. Nonetheless, several limitations should be acknowledged. First, the follow-up period was restricted to one week after the final treatment session, which limits conclusions about the long-term efficacy of the interventions. Second, although strict protocols were implemented to maximize participant and outcome assessor blinding, the physical distinction between the mechanical sensation of dry needling and the fluid expansion of an injection cannot be completely masked. This remains an inherent limitation of the study design, as the treating clinician could not be blinded to the procedure type; however, strict clinical blinding was successfully preserved regarding the specific type of anesthetic agent administered. Additionally, the absence of a true placebo control group and the reliance on self-reported outcome measures mean that our results may be influenced by individual perception, expectancy effects, and reporting bias. Although instruments like the Fonseca Anamnestic Index and Beck Depression Inventory are primarily screening tools, their established reliability for monitoring symptom progression and their high correlation with clinical indices justify their use for evaluating treatment response in this trial. Future studies should incorporate longer follow-up intervals, larger sample sizes, objective clinical assessments, and potentially multimodal approaches to better inform treatment strategies for myofascial pain.

From a clinical perspective, these findings provide flexible options for patient-centered management. Mepivacaine may be preferred when pronounced short-term analgesia is the primary objective. Nevertheless, lidocaine remains a highly accessible and effective pharmacological alternative. Furthermore, dry needling represents a valuable option in clinical contexts where a non-pharmacological approach is preferred or where the use of local anesthetics is contraindicated. Future studies should investigate cost-effectiveness and multimodal strategies to better integrate these modalities into routine clinical decision-making.

## Conclusions

In conclusion, dry needling, lidocaine, and mepivacaine are all effective short-term interventions, evaluated at a one-week follow-up, for managing myofascial pain of the masticatory muscles, yielding comparable improvements in self-reported TMD-related symptoms and depressive scores. Regarding pain intensity, while mepivacaine was the only intervention to statistically differentiate from the dry needling control group, no significant difference was observed directly between the two local anesthetics. Therefore, claims of clinical superiority for any single pharmacological agent should be interpreted with caution. While the pronounced numerical improvements associated with mepivacaine may theoretically align with its pharmacodynamic profile (such as efficacy without a vasoconstrictor), our study design precludes mechanistic conclusions. Ultimately, both lidocaine and mepivacaine represent highly viable, well-tolerated clinical options. Treatment selection should remain individualized, and future studies incorporating larger sample sizes, objective mechanistic data, and extended follow-up periods are necessary to firmly establish long-term comparative advantages.

## Data Availability

All data generated or analyzed during this study are included within this published article and its supplementary information files. Additional data are available from the corresponding author on reasonable request.

## Abbreviations

VAS: Visual Analog Scale
TMD: Temporomandibular Disorder
